# Shielding Effect of Escherichia coli O-Antigen Polysaccharide on J5-Induced Cross-Reactive Antibodies

**DOI:** 10.1128/mSphere.01227-20

**Published:** 2021-01-27

**Authors:** Pascal Rainard, Maryline Repérant-Ferter, Christophe Gitton, Pierre Germon

**Affiliations:** a INRAE, Université de Tours, UMR ISP, Nouzilly, France; U.S. Food and Drug Administration

**Keywords:** *Escherichia coli*, mastitis, J5 vaccine, opsonins, phagocytosis, cattle

## Abstract

Escherichia coli is the leading cause of severe mastitis in dairy farms. As E. coli mastitis is refractory to the hygienic control measures adapted to contagious mastitis, efficient vaccines are in demand. Existing mastitis vaccines, based on the use of killed rough E. coli J5 as the antigen, aim at inducing phagocytosis by neutrophils. We assessed the binding of J5-induced antibodies to isogenic rough and smooth strains along with a panel of mastitis-associated E. coli. Analysis by enzyme-linked immunosorbent assay revealed that antibodies to OmpA or killed J5 bind readily to rough E. coli but poorly to smooth strains. Flow cytometry analysis indicated that immunization with J5 induced antibodies that cross-reacted with rough E. coli strains but with only a small subpopulation of smooth strains. We identified type 1 fimbriae as the target of most antibodies cross-reacting with the smooth strains. These results suggest that the O-polysaccharide of lipopolysaccharide shields the outer membrane antigens and that only fiber antigens protruding at the bacterial surface can elicit antibodies reacting with mastitis-associated E. coli. We evaluated J5-induced antibodies in an opsonophagocytic killing assay with bovine neutrophils. J5 immune serum was not more efficient than preimmune serum, showing that immunization did not improve on the already high efficiency of naturally acquired antibodies to E. coli. In conclusion, it is unlikely that the efficiency of J5 vaccines is related to the induction of opsonic antibodies. Consequently, other research directions, such as cell-mediated immunity, should be explored to improve E. coli mastitis vaccines.

**IMPORTANCE** Despite intensive research, mastitis remains an important disease in dairy cattle with a significant impact on animal welfare, use of antibiotics, and, in the end, the economy of dairy farms. Although vaccines available so far have shown limited efficacy against coliform mastitis, vaccination is considered one of the measures that could limit the consequences of mastitis. One reason for the lack of efficiency of current vaccines likely stems from the current evaluation of vaccines that relies mostly on measuring antibody production against vaccine antigens. This report clearly shows that vaccine-induced antibodies fail to bind to most mastitis-associated E. coli strains because of the presence of an O-antigen and, thus, do not allow for improved phagocytosis of pathogens. As a consequence, this report calls for revised criteria for the evaluation of vaccines and suggests that cell-mediated immunity should be targeted by new vaccinal strategies. More generally, these results could be extended to other vaccine development strategies targeting coliform bacteria.

## Abstract (precis)

Despite intensive research, mastitis remains an important disease in dairy cattle with a significant impact on animal welfare, use of antibiotics, and, in the end, the economy of dairy farms. Although vaccines available so far have shown limited efficacy against coliform mastitis, vaccination is considered one of the measures that could limit the consequences of mastitis.

## INTRODUCTION

Mastitis, i.e., infection of the mammary gland with clinical symptoms, is the most prevalent disease of dairy cows worldwide (1, 2). Escherichia coli and other coliform bacteria are responsible for most of the severe mastitis cases affecting dairy herds despite the implementation of standard mastitis prevention programs (3, 4). This situation has prompted a sustained interest in coliform mastitis vaccines, which aim at reducing economic losses and the use of antimicrobials and at improving animal welfare. A few vaccines have been developed that provide some protection against mammary gland infections by coliforms (mainly E. coli and *Klebsiella* spp.). Those vaccines are based on the use of killed rough Gram-negative bacteria, particularly E. coli J5 (5, 6). Although they tend to reduce the severity of infections and consecutive milk losses, they do not decrease the incidence of infections. The protection afforded by E. coli J5 vaccines has been ascribed to enhanced concentrations in serum and mammary secretions of IgG and IgM antibodies that are directed toward conserved antigens on the surface of coliform bacteria (7, 8). Attempts to improve the efficacy of these vaccines have been directed at the production of higher concentrations of opsonic antibodies, which in the cow are the cytophilic IgM and IgG2 isotypes (9, 10).

The rationale behind the use of rough strains of Gram-negative bacteria is their capacity to induce cross-reactive antibodies directed at shared antigens, particularly those of the outer membrane. It has been established that smooth strains tend to elicit mainly antibodies to the immune-dominant O-antigen (11). These antibodies are highly effective at opsonizing the cognate strains (12), but they are serotype specific, and the O-serotype diversity of mammary gland-associated E. coli strains (MAEC) makes the O-antigen inappropriate for vaccine development (13–15). This is why antigens shared by coliform bacteria, such as the outer membrane proteins (Omps), have been targeted by vaccination with rough E. coli, like the J5 strain that is used in the current E. coli mastitis vaccines (5, 16, 17). Purified Omps, such as OmpA or FecA, which are abundant integral proteins of the outer membrane, have also been used as antigens with promising or disappointing results (18–21).

Opinions differ about the opsonic role of the J5 vaccine-induced antibodies. The accessibility of outer membrane antigens to antibodies is a matter of debate. Despite abundant literature, there is no consensus on the antibody biological activities, possibly because of the use of different techniques and strains (22). This is an important issue, because the defense of the mammary gland against bacteria relies heavily on phagocytosis by neutrophils, a notion particularly true of coliform mastitis (23). Opsonic antibodies bridge the phagocyte immunoglobulin receptors to accessible antigens at the bacterial surface. Consequently, antibodies to antigens that cannot be accessed or antibodies embedded in a bacterial exopolysaccharide are not opsonic. Naturally acquired antibodies to E. coli are present in the serum and milk of most cows (9, 24). Those antibodies play an important role in the protection of the mammary gland by opsonizing most of the MAEC. Their concentration and effectiveness can be high enough to drown out the effect of homologous O-serotype immunization (25).

To tackle the issue of the opsonizing activity of J5 vaccine-induced antibodies we used J5, two other smooth strains along with their rough isogenic variants, and a panel of MAEC strains to measure antibody binding by enzyme-linked immunosorbent assay (ELISA) and flow cytometry. The latter technique allowed us to use live bacteria while minimizing manipulations susceptible to alter their surface properties. We present evidence that the O-antigen shields the antigens recognized by most of the J5 vaccine-induced antibodies, which precludes their opsonic activity.

## RESULTS

### The analysis of the binding of OmpA- and J5-induced antibodies onto E. coli by ELISA distinguished rough from smooth strains.

**FIG 1:**
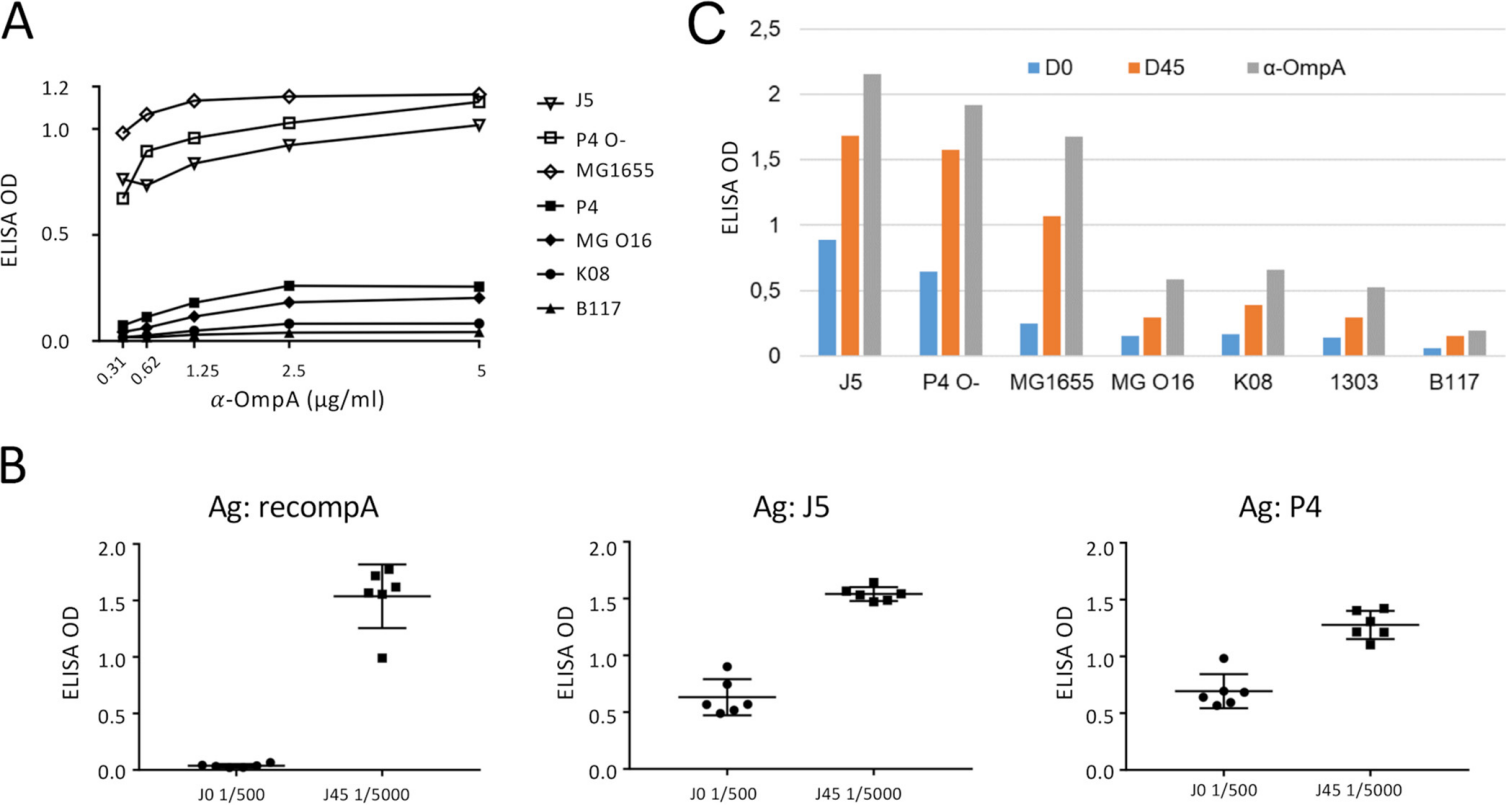
Binding of antibodies to E. coli rough and smooth strains, assessed by ELISA. (A) Different rough (J5, P4 O-, and MG1655) and smooth (P4, MG O16, K08, 1303, and B117) E. coli strains were used as antigens to measure the capacity of increasing concentrations of affinity-purified rabbit antibodies to recOmpA. (B) Assessment of the binding to recOmpA, J5, or P4 E. coli of 1/500 preimmune (D0) or 1/5,000 immune serum (D45) of 6 cows. Differences (before/after immunization with the 7 strains) are significant (*P = *0.03, Wilcoxon matched-pairs rank test). (C) Assessment of the binding of antibodies to OmpA (5 μg/ml) or J5 before (D0, 1/100) or after (D45, 1/5000) immunization to rough and smooth E. coli strains.

Affinity-purified antibodies to recOmpA bound readily to rough strains but hardly to smooth strains, even at high concentration (5 μg/ml) (Fig. 1A). As numerous copies of OmpA are inserted in the outer membrane of E. coli, this result suggests that antibodies to Omps are prevented from reaching their target by the O-antigen component of lipopolysaccharide (LPS). However, Omps other than OmpA are also present in large numbers in the outer membrane of Gram-negative bacteria, and vaccination with rough strains is supposed to induce antibodies to a variety of Omps. To be more inclusive, we immunized six cows with J5 bacteria and tested the obtained immune sera against OmpA, J5, and P4 strains of E. coli by ELISA (Fig. 1B). Pools of preimmune and immune anti-J5 sera were used to assess the reactivity of vaccine-induced antibodies with a panel of rough and smooth E. coli strains. The pooled immune sera were diluted 1/5,000 to drown out natural antibodies. The cows produced vaccine-induced antibodies that recognized recOmpA and the J5 strain, as expected, but also the P4 strain. The titers of antibodies to OmpA increased markedly, partly because there were hardly detectable natural antibodies before immunization. Naturally acquired antibodies to E. coli J5 and P4 were detected in the preimmune sera, an expected finding. Titer increases were high against J5 as expected, but sizable increases against P4 bacteria were also detected. That result was rather unexpected on the basis of the poor reactivity of antibodies to OmpA with smooth E. coli strains but compatible with the idea that the response to J5 cannot be reduced to the response to one surface antigen, even one that is highly expressed. Under this condition, there was an increase in the ELISA optical density (OD) following immunization with all the strains tested, but the binding of anti-OmpA or anti-J5 antibodies was markedly lower to smooth than to rough strains (Fig. 1C).

These results suggest that most of the J5-induced antibodies were prevented from reaching their antigen targets by the O-polysaccharide of smooth strains. Nevertheless, the ELISA used to assess the binding of antibodies to E. coli has some limitations. The manipulations required to adhere the bacteria to the ELISA plates and the heat treatment may have modified their surface properties, and smooth bacteria tend to adhere less than rough bacteria to the microtiter plates, biasing direct comparisons of ELISA OD values. Moreover, the measures are the means of individual bacteria that compose a bacterial population and cannot unveil possible bacterial heterogeneity. To obviate these limitations, we used flow cytometry analysis of live bacterial populations. This method is compatible with limited manipulations of bacteria and, thus, limited alterations of the bacterial surface and enables the analysis of a large number of individual bacteria, yielding a spectral image of the bacterial population (26).

### Bacterial flow cytometry reveals that J5-induced antibodies label only a minor subpopulation of smooth E. coli.

**FIG 2:**
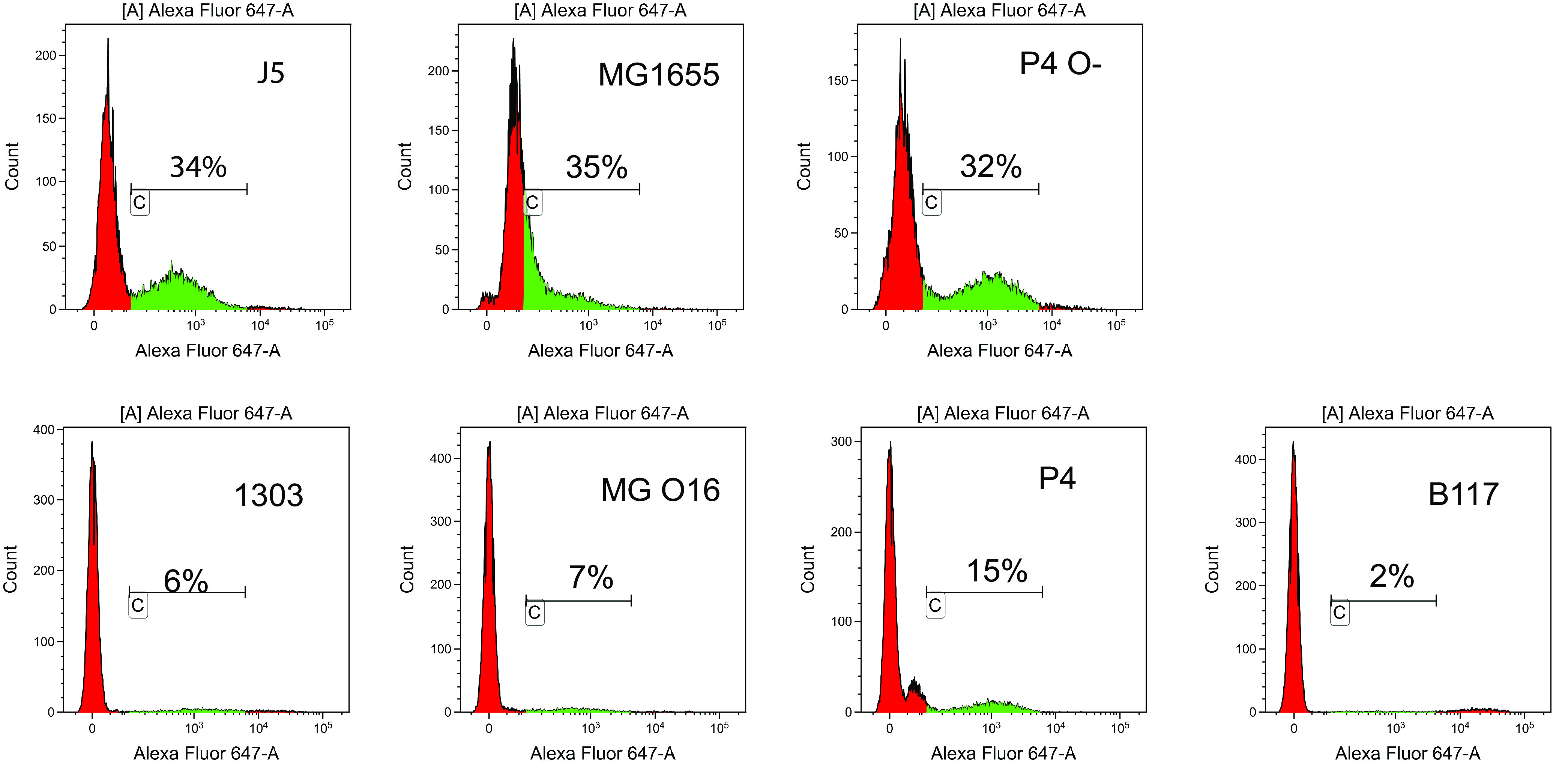
Analysis of the binding to rough (J5, MG1655, and P4 O-) and smooth (1303, MG O16, P4, and B117) E. coli strains of bovine antibodies to OmpA. Bacteria were incubated with affinity-purified bovine antibodies to recOmpA (5 μg/ml) and then with the secondary antibody conjugated to Alexa Fluor 647. The analysis was restricted to the bacteria within the gate of isolated bacteria, excluding the clumps. The indicated percentages are percentages of fluorescent bacteria. Results are from a representative experiment of at least three replicates per strain.

Nonspecific binding of antibodies to bacteria may impede the use of the flow cytometry technique, as it occurs with Staphylococcus aureus expressing protein A. Therefore, we first checked that the rough and smooth E. coli strains did not bind bovine antibodies by measuring the labeling of bacteria with affinity-purified antibodies to ovalbumin and the secondary antibody coupled to the fluorophore (Alexa Fluor 647). Only small numbers of bacteria were labeled, with some variation among strains (see Fig. S1A in the supplemental material). We then assessed the binding of affinity-purified bovine antibodies to OmpA. The gating strategy is depicted in Fig. S2. Preliminary titration experiments indicated a plateau of labeling at 2.5 g/ml; thus, a saturating concentration of 5 μg/ml was selected (Fig. S3). Labeling of bacteria was not uniform for a given strain, indicating heterogeneity in the bacterial suspension. Despite this, compared to smooth strains, a slight shift to the right of the emission spectra was observed with all rough strains along with a sizable proportion (about one-third) of the rough bacteria labeled with a mean fluorescence intensity above 100 (Fig. 2, gate C, in green). Among the smooth strains, consistent with the ELISA results, P4 cells were the most labeled bacteria but in a lower proportion than its rough isogenic mutant (15% versus 32%). The other rough-smooth pair of E. coli (MG1655 versus MG O16) showed the same trend, 7% versus 35% of labeling. The other two smooth strains tested (1303 and B117) were not labeled more than that with the anti-ovalbumin control.

10.1128/mSphere.01227-20.1 FIG S1 (A) Assessment of nonspecific antibody binding. Rough (J5, MG1655, and P4 O-) or smooth (B117, MG O16, and P4) E. coli strains were incubated with affinity-purified bovine antibodies to ovalbumin (5 μg/ml), washed, and incubated with the secondary antibody [anti-bovine IgG(H+L) coupled to Alexa Fluor 647] before analysis by flow cytometry. Percentages of nonspecific binding or noise of gated bacteria varied from 3.5 to 10%. (B) Bacteria were incubated with J5 immune serum (1/1,000) and the secondary antibody. All the rough bacteria (P4 O-) were labeled, and only a proportion of the parent smooth strain showed the same intensity of labeling. This proportion was similar when bacteria were used at the stationary (P4) or the exponential (P4 expo) phase of growth. (C) The strain P4 was incubated with anti-J5 antibody and labeled with secondary antibody. Propidium iodide (PI) was added before analysis by flow cytometry. Dead bacteria appear in green and anti-J5-labeled bacteria in dark blue. The figure shows that the antibody-labeled bacteria were not dead. Download FIG S1, PDF file, 0.2 MB. Copyright © 2021 Rainard et al. 2021 Rainard et al. This content is distributed under the terms of the Creative Commons Attribution 4.0 International license.

10.1128/mSphere.01227-20.2 FIG S2 Gating and analysis of labeling of bacteria incubated with 5 μg/ml antibodies to OmpA. The forward scatter (FSC)/side scatter (SSC) dot plot shows that the bacteria labeled by the antibodies (green dots and gate B) appear slightly larger (FSC) and more irregular (SSC), likely in relation to a tendency to aggregate. Unlabeled or faintly labeled bacteria appear red. Download FIG S2, PDF file, 0.06 MB. Copyright © 2021 Rainard et al. 2021 Rainard et al. This content is distributed under the terms of the Creative Commons Attribution 4.0 International license.

10.1128/mSphere.01227-20.3 FIG S3 Titration of antibodies to OmpA by flow cytometry. Increasing concentrations of antibodies to OmpA (0.625, 1.25, and 2.5 μg/ml) were used to label rough strains (J5 and P4 O-) or smooth strains (P4 and MG O16) of E. coli. Two populations of bacteria were identified, one clearly labeled and another either faintly labeled (rough strains) or not labeled (smooth strains). Download FIG S3, PDF file, 0.2 MB. Copyright © 2021 Rainard et al. 2021 Rainard et al. This content is distributed under the terms of the Creative Commons Attribution 4.0 International license.

**FIG 3:**
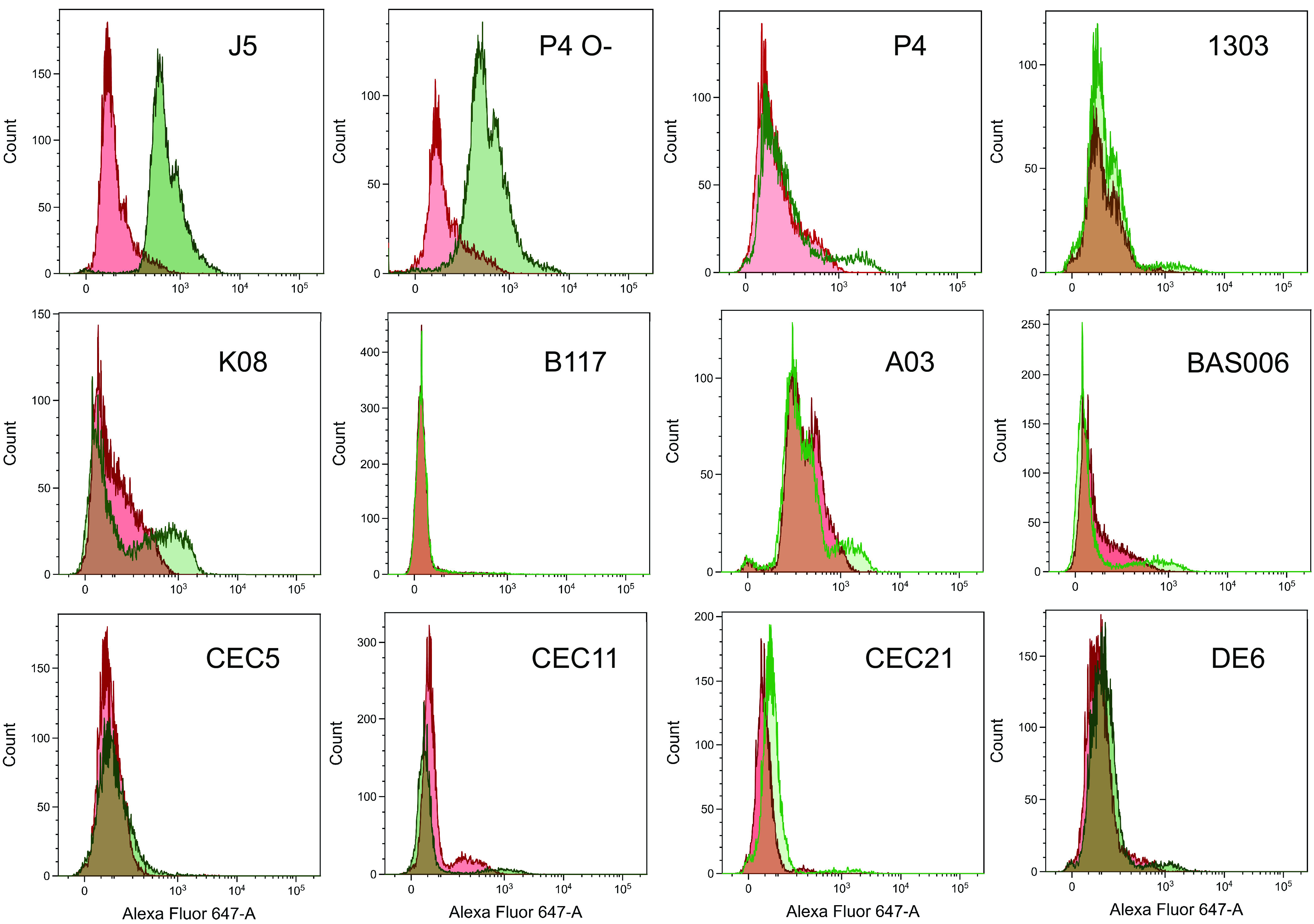
Analysis of the binding of J5 vaccine-induced antibodies to rough E. coli strains (J5 and P4 O-) and smooth E. coli strains. Pooled preimmune sera and immune sera were used diluted at 1/500. The red histogram in the overlays shows the bacterial distribution with preimmune serum, and the green histogram shows the bacterial distribution with the immune serum.

We then assessed the capacity of the J5 immunization to induce antibodies to MAEC strains. To this end, we compared the labeling of the strains with preimmune and immune serum (pooled from the immunized cows), at a dilution (1/500) that did not completely drown out natural antibodies, as shown by the labeling of all the bacteria (except strain B117) by the preimmune serum (Fig. 3). After immunization with J5 bacteria, the pool of immune bovine serum labeled the J5 and P4 O- rough strains more strongly than did the preimmune serum, showing only one rather homogeneous population of bacteria (Fig. 3). All rough bacteria reacted with the vaccine-induced antibodies, a result in keeping with their cross-reactive quality. When smooth strains were analyzed, it appeared that in all cases a majority of the bacterial populations were not more labeled by the immune than by the preimmune serum (Fig. 3). A few strains did not show any increase in labeling, such as the encapsulated strain B117 or the MAEC strain CEC5 or CEC21, whereas the other strains, such as P4, showed a small subpopulation that was labeled by the immune serum. This may explain the increase in the P4 ELISA OD after immunization (Fig. 1C).

**FIG 4:**
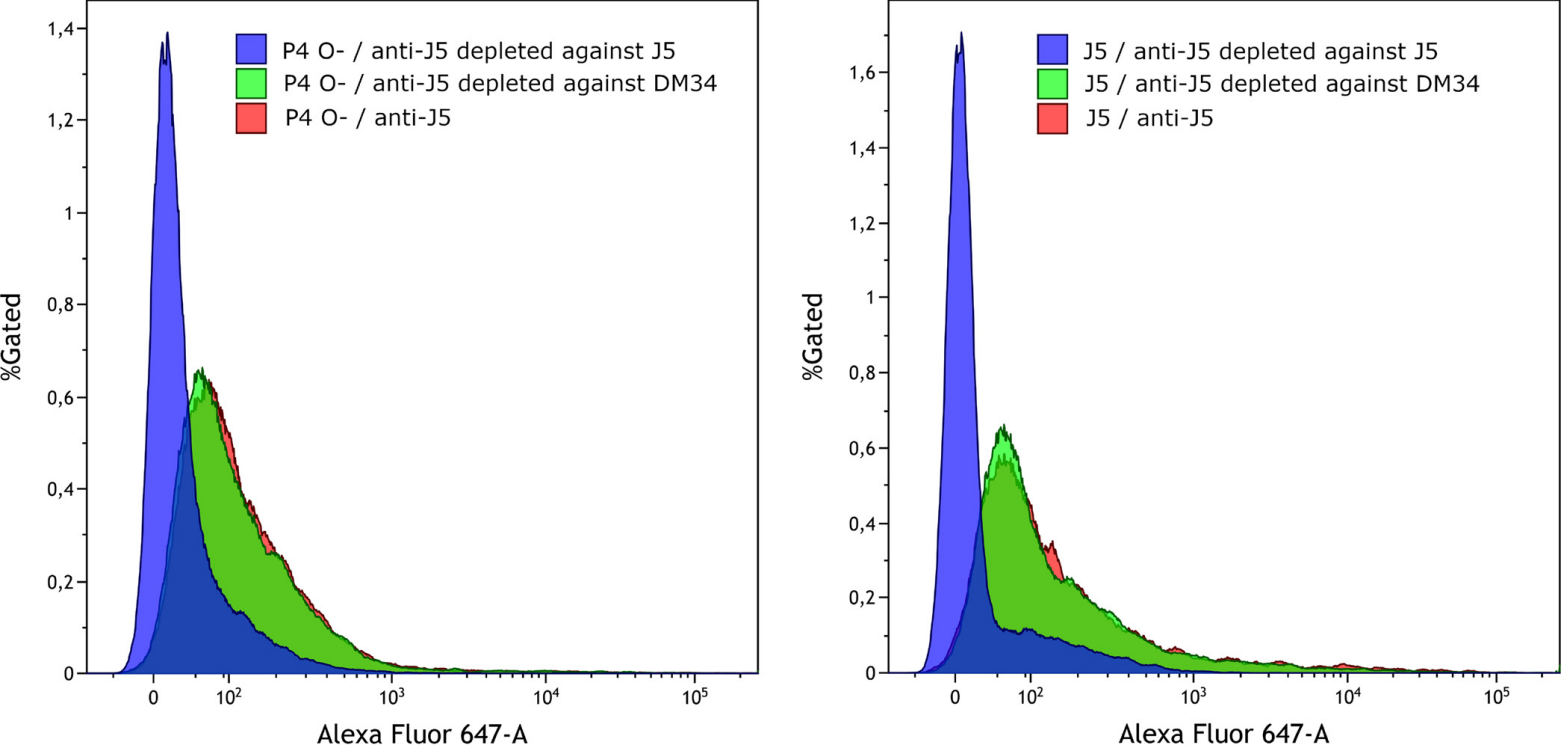
Analysis labeling of rough E. coli strains P4 O- (left) and J5 (right) with the nondepleted mock-treated J5 serum (red) or with J5 immune serum depleted by adsorption with the rough E. coli strain J5 (blue) or the smooth strain DM34 (green). Sera were used diluted at 1/500.

To further investigate the weak labeling of smooth strains by the J5 antiserum, our rationale was that if outer membrane proteins from smooth strains were accessible to antibodies present in the J5 antiserum, adsorption of the J5 antiserum with smooth bacteria should remove these antibodies and reduce the labeling of these same outer membrane proteins on the surface of rough strains. We adsorbed the anti-J5 serum with the smooth strain DM34. As controls, the J5 serum was mock treated (incubation with phosphate-buffered saline [PBS] without bacteria) or adsorbed with J5 rough bacteria. These sera were then used to label rough strains P4 O- and J5. Results indicate that adsorption with strain DM34 did not modify the labeling of strains J5 and P4 O- (Fig. 4, compare red versus green histograms). On the contrary, as expected, adsorption of J5 antiserum with the rough strain J5 prevented labeling of J5 bacteria (Fig. 4, blue histogram).

We then speculated that the reaction of immune serum with only a subpopulation of smooth bacteria could result from the interaction of antibodies with antigens protruding from the LPS O-antigen layer and expressed only by a fraction of a bacterial population. Type 1 fimbriae are such candidate antigens, as they protrude from the surface of bacteria and are usually expressed by only a small proportion of bacteria in a population, including MAEC strains (27–29).

### Immunization with J5 elicits cross-reactive antibodies to type 1 fimbriae.

**FIG 5:**
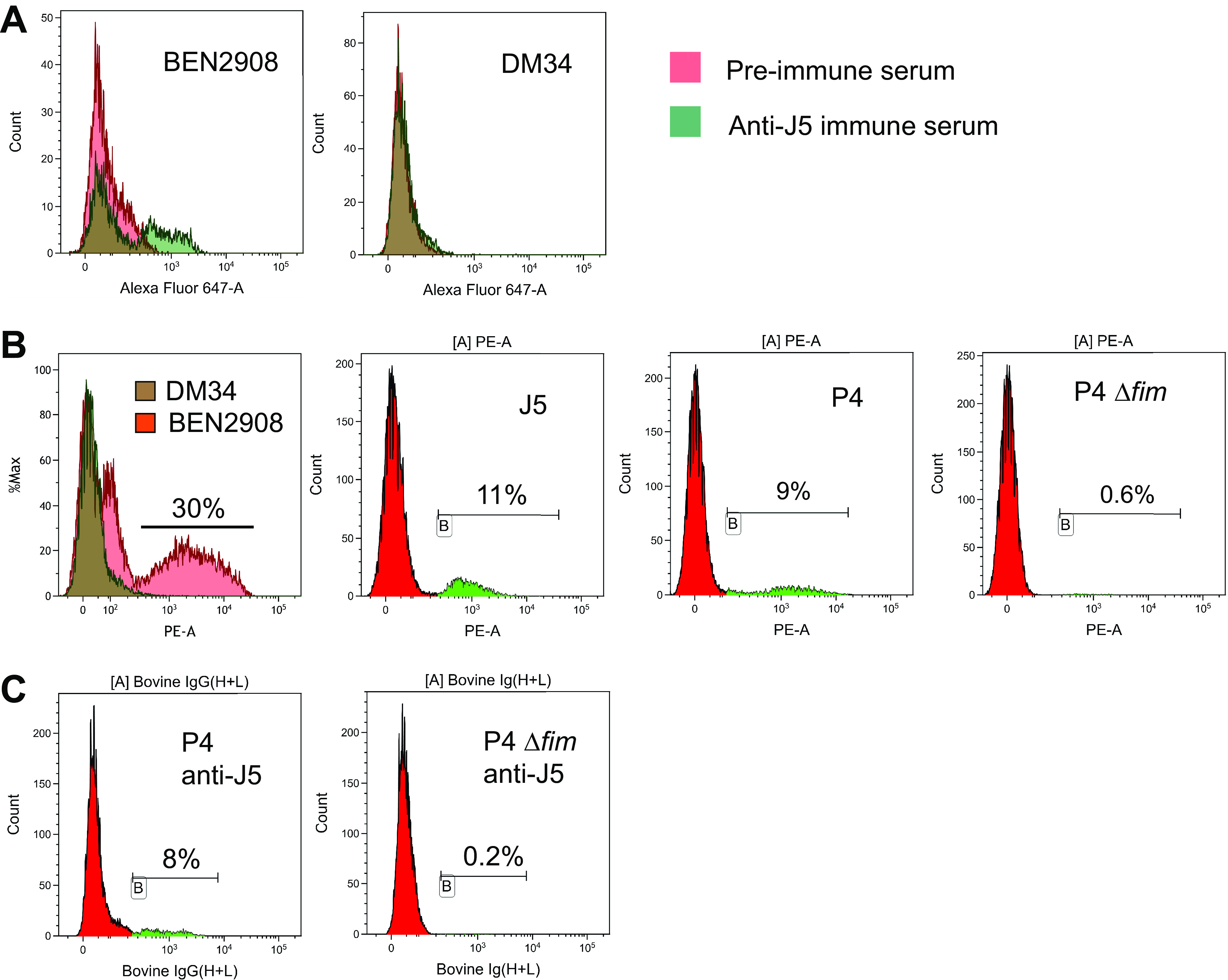
(A) Binding of preimmune (red histogram) or immune (green histogram) serum (1/2,000) to J5 to E. coli BEN2908 (producer of type 1 fimbriae) or to its Δ*fim* mutant DM34. (B) Binding of rabbit antiserum (1/100) to type 1 fimbriae to E. coli. (C) Binding of anti-J5 immune serum (1/2,000) to E. coli P4 or to its Δ*fim* mutant (P4 Δ*fim*).

To test the fimbria hypothesis, we first checked if the J5 vaccine elicited antibodies to type 1 fimbriae. To this end, we used a type 1 fimbria-producing strain (BEN2908) and its mutant deficient in type 1 fimbriae (DM34). After immunization with J5, a sizable proportion (20% to 30%) of BEN2908 bacteria was labeled, contrary to the mutant bacteria (Fig. 5A). We then used a rabbit antiserum to type 1 fimbriae to visualize the production of fimbriae by E. coli under our culture and analysis conditions. This serum labeled 30% of the type 1 fimbria-producing strain BEN2908 and did not label the defective mutant (Fig. 5B). It labeled 11% of J5 bacteria, showing that this strain can produce type 1 fimbriae, in keeping with its capacity to elicit antibodies to this bacterial component (Fig. 5B). Nine percent of P4 bacteria were labeled but hardly any bacteria of the P4 Δ*fim* type 1 fimbria-defective mutant. Finally, we analyzed the binding of anti-J5 antibodies to P4 and its type 1 fimbria-defective mutant. About 8% of P4 bacteria bound the anti-J5 immune serum, a percentage similar to the 9% of bacteria labeled with the rabbit anti-type 1 fimbriae (Fig. 5B and C). The P4 Δ*fim* defective mutant did not bind the anti-J5 serum (Fig. 5C). These results suggest that most of the J5-induced antibodies that cross-react with P4 were directed to type 1 fimbriae.

**FIG 6:**
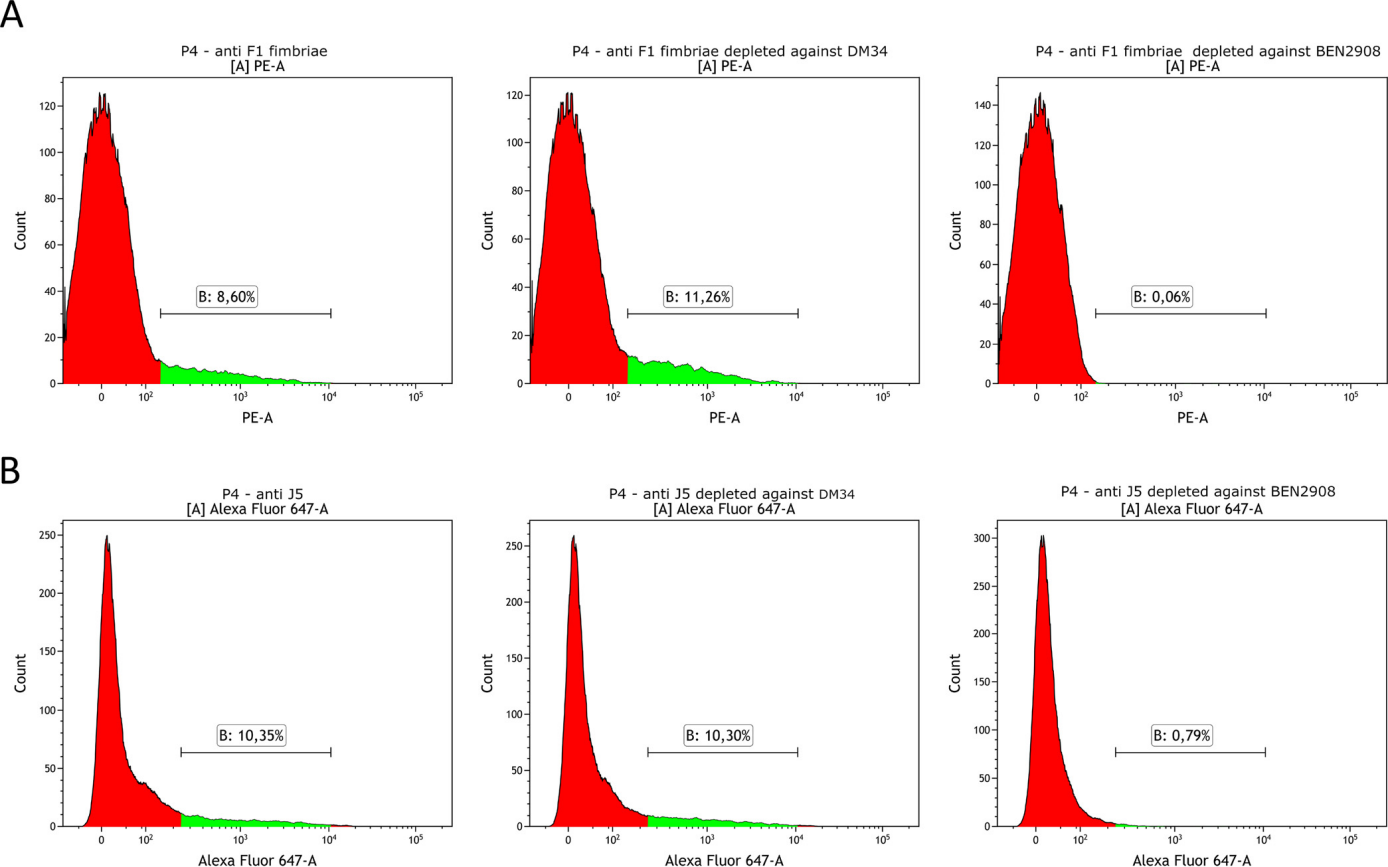
Labeling of E. coli strain P4 with antisera depleted with type 1 fimbriae. Anti-type 1 fimbriae (A) and anti-J5 serum (B) were adsorbed with the smooth strain BEN2908 or with the isogenic Δ*fim* mutant DM34. As a control, antisera were mock treated by incubation in PBS without bacteria. Sera were used diluted at 1/500. Bacteria were then incubated with anti-rabbit PE-coupled IgG antibody (A) or Alexa647-coupled anti-bovine IgG+IgM antibody. The green area on each histogram indicated labeled bacteria.

To confirm that the proportion of P4 bacteria labeled with the J5 antiserum was linked to the expression of type 1 fimbriae by these bacteria, we depleted the J5 antiserum from antibodies recognizing type 1 fimbriae by adsorption with strain BEN2908 and repeated the labeling of P4 bacteria (Fig. 6). The capacity of the adsorption method to deplete type 1 fimbria antibodies was verified by adsorption of type 1 fimbria antiserum with strain BEN2908 and its Δ*fim* mutant, DM34 (Fig. 6A). Compared to the mock-treated serum, adsorption with strain BEN2908 significantly reduced the labeling of strain P4, while labeling was not modified after adsorption with strain DM34. We then proceeded to the analysis of P4 labeling with J5 antiserum after adsorption with strains BEN2908 and DM34. As expected, we observed a loss of labeling of P4 with the serum adsorbed with strain BEN2908 and not with its isogenic mutant, DM34 (Fig. 6B).

### J5-induced antibodies do not improve the phagocytic killing of MAEC strains.

An important activity of antibodies and complement concerning mammary gland infections is the opsonization of bacteria to allow phagocytosis and killing of bacteria by neutrophils. Our phagocytic killing assay involved the opsonization of bacteria with J5-induced antibodies and incubation with activated neutrophils, activation being achieved by the use of precolostral calf serum (PCCS) as a source of complement.

We first checked the resistance of E. coli strains to the bactericidal activity of complement from PCCS in the presence or absence of J5-induced antibodies (Table S1). The rough strains P4 O- and MG1655 were killed by 5% PCCS, whereas the isogenic smooth strains P4 and MG O16 resisted 80% PCCS. Even in the presence of antibodies, the smooth strains resisted the bactericidal effect of high concentrations of normal bovine serum, taking into account the agglutination of bacteria by antibodies and conglutinin (Table S2).

10.1128/mSphere.01227-20.5 TABLE S1 Resistance of E. coli strains to the bactericidal activity of complement. Figures are numbers of CFU after 3 h of incubation of bacteria at 37°C with the indicated final concentrations of serum. PCCS was used as a source of complement almost devoid of antibodies. ND, not done. Download Table S1, DOCX file, 0.01 MB. Copyright © 2021 Rainard et al. 2021 Rainard et al. This content is distributed under the terms of the Creative Commons Attribution 4.0 International license.

10.1128/mSphere.01227-20.6 TABLE S2 Resistance of smooth E. coli strains to the bactericidal activity of normal bovine serum. Shown are numbers of CFU after 3 h of incubation of bacteria at 37°C with the indicated final concentrations of serum. SBN was used as a source of complement and antibodies. Sonication was used to disperse the bacteria agglutinated by the antibodies and conglutinin. Download Table S2, DOCX file, 0.01 MB. Copyright © 2021 Rainard et al. 2021 Rainard et al. This content is distributed under the terms of the Creative Commons Attribution 4.0 International license.

We also used flow cytometry to confirm the absence of antibodies to E. coli in PCCS as well as the effectiveness of PCCS at a concentration of 10% for the deposition of C3 fragments onto bacteria (Fig. S4). Effective deposition of C3 was observed with rough E. coli and MAEC P4 but not MAEC 1303, whereas antibodies to E. coli were hardly detectable.

10.1128/mSphere.01227-20.4 FIG S4 Assessment by flow cytometry of the occurrence of antibodies to E. coli in PCCS and the deposition of complement onto bacteria. Bacteria were incubated for 90 min with 10% PCCS, labeled for the binding of C3 fragments (C3b/C3bi) and antibodies (IgG and IgM) at their surface (see Materials and Methods), and analyzed by flow cytometry. Download FIG S4, PDF file, 0.2 MB. Copyright © 2021 Rainard et al. 2021 Rainard et al. This content is distributed under the terms of the Creative Commons Attribution 4.0 International license.

**FIG 7:**
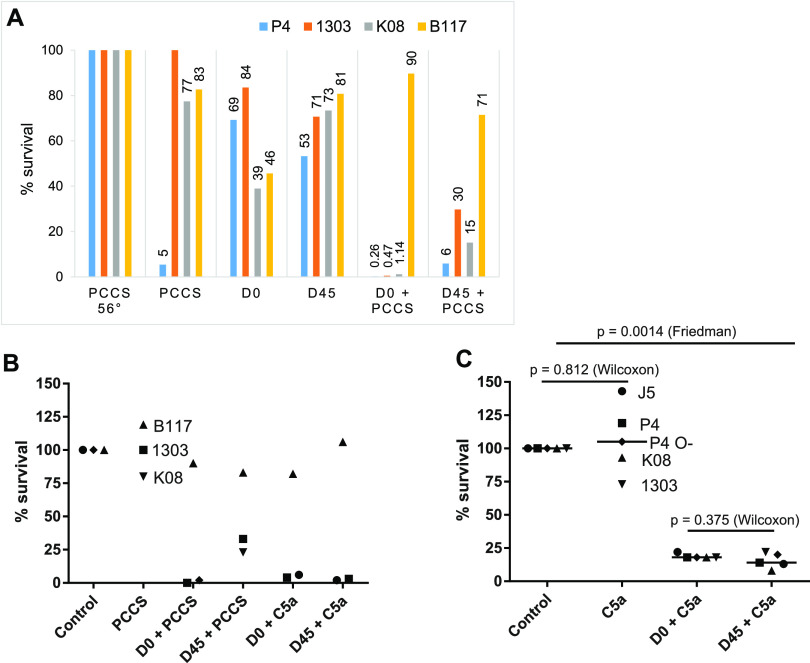
(A) Percent survival of E. coli strains (P4, 1303, K08, and B117) after 90 min of exposure to PMNs and a source of complement (10% PCCS) with or without 0.2% preimmune (D0) or immune (D45) serum to J5 with or without 10% PCCS. (B) Percent survival of E. coli strains (B117, 1303, and K08) after 90 min of exposure to PMNs and 0.2% preimmune (D0) or immune (D45) serum to J5 with or without 10 nM C5a. (C) Percent survival of E. coli strains (P4, 1303, K08, J5, and P4 O-) after 90 min of exposure to PMNs activated by 10 nM C5a with either 0.2% preimmune (D0) or immune (D45) serum to J5.

Despite C3 fragment deposition onto bacteria, PCCS alone was not sufficient to allow the killing by granulocytes, except for strain P4, which was susceptible to phagocytic killing in the presence of PCCS only (Fig. 7A). Antibodies alone (0.2% serum) did not allow the polymorphonuclear neutrophils (PMNs) to kill the bacteria efficiently, and there was no improvement with the immune serum (Fig. 7A). The combination of antibodies and PCCS was necessary to achieve a strong killing of all strains but B117. Again, the immune serum was not more efficient than the preimmune serum (Fig. 7A). There was even a trend toward a reduced opsonic activity of the immune serum in the presence of PCCS.

Because the activity of PCCS in the phagocytic killing assay could be linked to the deposition of C3 fragments (C3b and C3bi) onto the bacteria or to the generation of PMN-activating molecules, such as C5a, we performed phagocytic killing assays, replacing PCCS with purified bovine C5a. In combination with antibodies, C5a alone actually was sufficient to activate PMNs and to allow PMNs to kill E. coli P4, K08, or 1303 but not the encapsulated strain B117 (Fig. 7B). This showed that the deposition of C3 fragments was not necessary when MAECs were opsonized with antibodies, and the PMNs were activated. Similar to the results obtained with PCCS, there was no difference between the opsonic activity of preimmune and immune sera, even with the rough strains J5 and P4 O- (Fig. 7C). Altogether, these results indicate that the J5-induced antibodies did not improve on the naturally acquired antibodies present in normal bovine serum.

## DISCUSSION

The point at issue is the capacity of antibodies elicited by vaccination with a rough E. coli strain such as J5 to induce protection against mastitis by coliform bacteria. Currently, the J5 vaccines aim at inducing opsonic antibodies, and attempts to optimize the immune response are measured through increases in antibody titers (7, 30, 31). This is in keeping with the importance of phagocytosis by neutrophils as an essential defense of the mammary gland against E. coli (23). We examined the validity of this approach by assessing whether J5-induced antibodies could opsonize MAEC strains. Since a prerequisite is that the induced antibodies can bind to their bacterial antigen to bridge the bacteria to the phagocytes, we analyzed the interaction of vaccine-induced antibodies with a panel of E. coli rough and smooth strains. We confirmed that immunization of cows with J5 bacteria elicits antibodies that cross-react with rough E. coli strains (Fig. 3), in accordance with early studies showing that antibodies to Omps are induced (32). Nevertheless, very few bacteria within populations of smooth E. coli strains were labeled by the immune serum when naturally acquired antibodies were watered down (Fig. 3). This is exemplified by antibodies to a major Omp, OmpA (Fig. 1A and 2). Results obtained by ELISA with heat-killed bacteria and by flow cytometry with live bacteria concurred with this conclusion. The use of pairs of rough and smooth isogenic strains indicated that the O-antigen was responsible for the low reactivity of most smooth bacteria with the vaccine-induced antibodies. In addition, adsorption of the J5 antiserum with smooth bacteria did not modify the labeling of rough strains (Fig. 4), indicating that outer membrane proteins of smooth strains were not readily accessible to adsorb antibodies recognizing these proteins in the J5 antiserum.

**FIG 8:**
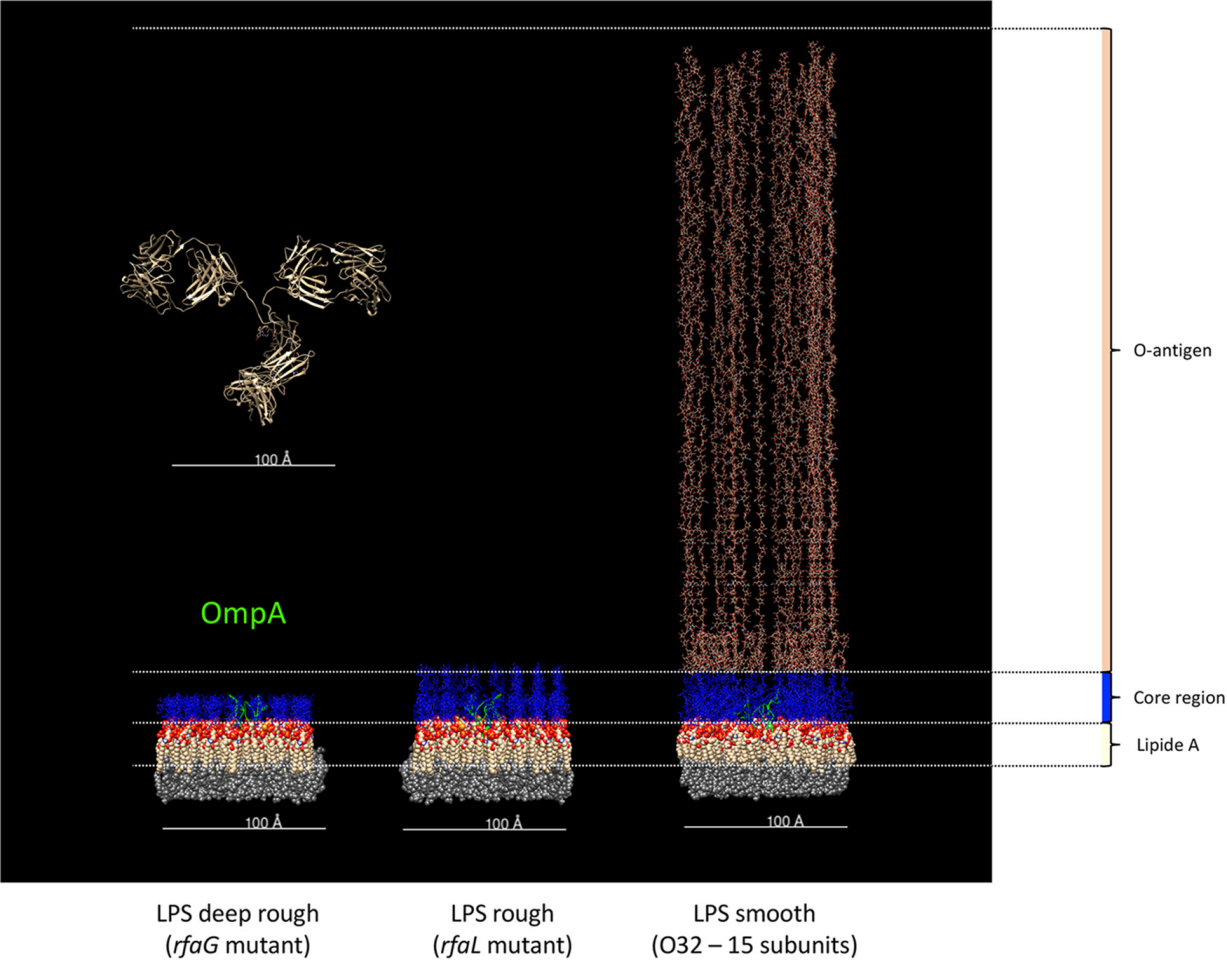
E. coli outer membrane containing LPS molecules with different numbers of O32 antigen subunits and one OmpA molecule (PDB entry 1BXW) was modeled using CHARMM (http://charmm-gui.org/) and processed using Chimera (48). The lipid A moieties are shown as red and beige balls, while the core LPS is shown as blue chains and the O32 repeats as orange-beige chains. An IgG molecule (PDB entry 1IGT) is shown to scale for comparison.

These findings are reminiscent of earlier results with monoclonal antibodies (MAbs) to porins showing the absence of binding to the surface of bacteria possessing an O-antigen (33). Modeling of the outer leaflet of the E. coli outer membrane along with an IgG antibody suggests that binding of antibodies to outer membrane proteins is impeded by steric hindrance due to the presence of the O-antigen (Fig. 8). Flow cytometry analysis of antibody-labeled bacteria was very efficient at characterizing the phenotypic heterogeneity of the E. coli populations in terms of reactivity with OmpA-induced antibodies as well as with J5-induced antibodies. Although we have no precise explanation at this time, these results are reminiscent of the heterogeneity of bacterial cultures unraveled by single-cell analyses using flow cytometry (34). The obtained results prompted us to look for the surface antigens responsible for the labeling by anti-J5 antibodies of the small subpopulations.

By using antibodies specific to type 1 fimbriae and defective mutants, we showed that antibodies to type 1 fimbriae could account for most, if not all, of the J5-induced antibodies that react with MAEC strains (Fig. 5). Furthermore, when J5-induced antibodies were adsorbed with the type 1 fimbriated strain BEN2908, the labeling of P4 was almost fully abolished, while it was unaffected when these antibodies were adsorbed with strain DM34, the Δ*fim* derivative of strain BEN2908 (Fig. 6). This suggests that antigens that protrude from the LPS O-antigen layer can be targets of vaccine-induced cross-reactive antibodies. Type 1 fimbriae are produced by commensal and pathogenic E. coli strains, and they contribute to the invasion of epithelial cells (27). Fimbriae are produced by some MAECs (28). This expression can be much higher in the host environment (infection niche) than under *in vitro* conditions, but only a proportion of the bacteria express fimbriae at a given time (35). Fimbriae may promote adhesion to bovine mammary epithelial cells, but adhesion is inhibited by milk (36). The production of adhesins may expose bacteria to phagocytosis, although many pathogenic bacteria protect themselves by also producing an antiphagocytic polysaccharidic surface layer (37). The opsonic efficiency of antibodies to fimbriae remains to be established.

We investigated the opsonic activity of J5-induced antibodies by comparing the capacity of preimmune and immune sera to promote the phagocytic killing of rough and smooth strains. Adult cows possess naturally acquired antibodies to MAECs, which are difficult to water down while keeping sufficient concentrations of vaccine-induced antibodies. Serum concentrations appropriate to discriminate preimmune from immune sera are low, less than 1% for phagocytosis (25) or much lower when sensitive assays are used (ELISA or flow cytometry), as in the present study. At these low concentrations, complement is not active; this is why we added either PCCS or C5a to the assay. In the presence of C5a, phagocytic killing was optimal, as previously reported (38). We could not show an advantage of immune over preimmune serum in terms of bacterial killing by PMNs (Fig. 7). Although the minor bacterial subpopulations expressing type 1 fimbriae may have been opsonized, the results indicate that the bulk of the population was refractory to the opsonic effect of J5-induced antibodies.

Complement deposition onto the bacteria (see Fig. S2 in the supplemental material) during the phagocytosis assay with PCCS did not make the smooth strains more susceptible to the immune serum. This result excludes the possibility of permeability increase of the O-antigen layer by complement fragment insertion or enzymatic effect, as suggested to explain the activity of antibodies to Omps (39). It appeared that complement deposition onto bacteria was not necessary even at the low serum concentration used (0.5%), provided that the phagocytes were activated by C5a. Complement in milk can exert its bactericidal activity (40), but MAECs are serum resistant, with the O-antigen contributing to this resistance (41). Our results also confirm that naturally acquired antibodies were efficient at opsonizing MAEC strains, in accordance with previous reports (9, 42). The resistance to phagocytosis of encapsulated strains such as B117 has already been documented (24) and conforms to the requirement for antibodies to K antigen for phagocytosis to be efficient (12). Our results are in keeping with the idea that the main opsonic activity is mediated by antibodies to the O-antigen (12, 22).

In conclusion, our results are not in favor of the use of J5 bacteria to improve the opsonic activity of serum or milk through vaccination. Natural antibodies, mainly IgG and IgM in cow milk, are present in milk in sufficient concentrations to opsonize efficiently most MAECs, and this opsonic activity increases during mastitis thanks to plasma exudation that drives antibodies to the mammary gland lumen (24, 25, 43). Nevertheless, antibodies to extracellular fibers such as fimbriae or flagella may play a role in the defense of the mammary gland against coliform infections, possibly by interfering with adhesion to mammary epithelial cells or the spreading of the bacterin in the lumen of the mammary gland. Moreover, the humoral response is not the only immune response that could be induced by Omps and that could be beneficial to the host. Cell-mediated immune responses, a neglected area of research in the mastitis field until recently, may prove to be a promising lead to the development of new vaccines (44). In this view, antibody titers may not be a good correlate of protection, particularly those antibodies that bind to rough E. coli strains. Alternatively, measuring cell-mediated responses, such as the production of cytokines by blood cells stimulated by E. coli or E. coli antigens, may offer new prospects.

## MATERIALS AND METHODS

### Ethics statement.

Animal experiments were conducted in compliance with all applicable provisions established by the European Union directive 2010/63/UE. Blood sampling was approved by the Ethics Committee of Val de Loire (agreement no. 4809 INRA) under agreement APAFIS#4809-2019050318255469. Sampling was performed by authorized staff members of the permanent dairy herd of the INRA Experimental Unit UE-PAO (agreement no. F37-175-2; Nouzilly, France) in strict accordance with good clinical practices. Anti-J5-OmpA immunization was performed at the INRA experimental farm of Bressonvilliers under agreement APAFIS#11503-2017091411167913.

### Nonimmune and immune sera.

Fresh pooled normal bovine serum (NBS) was obtained from 12 Holstein cows (in first or second lactation), allowed to clot at 37°C for 2 h, centrifuged at 2,500 × *g* for 20 min, and stored in portions at –80°C. Precolostral calf serum (PCCS) was prepared similarly from a blood sample from a newborn unsuckled calf. Complement was inactivated when required by heating sera at 56°C for 30 min (H-NBS and H-PCS). Immune serum to J5 was obtained from six cows immunized with 2 × 10^9^ heat-killed (60°C for 45 min) J5 bacteria complemented with 20 μg recombinant E. coli OmpA (recOmpA) emulsified in oily adjuvant (Montanide ISA 61G; Seppic) administered twice at 4-week intervals by the intramuscular route. recOmpA was obtained as described previously (18). Affinity-purified antibodies to recombinant E. coli OmpA (recOmpA) were prepared from the serum of cows immunized with recOmpA as described for the preparation of rabbit antibodies to OmpA (18). Rabbit antisera to F1 fimbriae were obtained by intramuscular injection of 250 μg of purified fimbriae and were described in a previous publication (45).

### Escherichia coli strains.

The E. coli strains used in this study and their phenotypes are listed in Table 1. This panel of strains included theP4/P4 Δ*rfb* and MG1655 O16/MG1655 pairs of isogenic smooth (S) and rough (R) strains. Smooth strains representative of MAECs, chosen to cover the diversity of phylogroups and core types found among MAECs, were also used in this study. The P4 Δ*fim* strain was obtained from strain P4 by the method of Datsenko and Wanner using primers PG549_del_op_fim (GAACGACTGCCCATGTCGATTTAGAAATAGTTTTTTTAAAGGAAAGCAGCGTGTAGGCTGGAGCTGCTTC) and PG550_del_op_fi (TAGCTTCAGGTAATATTGCGTAACTGCATTAGCAATGCCCTGTGATTTCTCATATGAATATCCTCCTTAG) (46).

**TABLE 1:** E. coli strains and mutants used in the study

Strain	Pathotype/genotype^*a*^	Phylogroup/core type	Serotype	Phenotype	Reference or source
J5		/R3	ΔO111	R (Rc)	
MG1655		A/K-12		R	
MG O16	MG1655 *wbbL*^+^	A/K-12	O16	S	49
P4	MAEC	A/K-12	O32:H37	S	50
P4 O-	P4 Δ*rfb*	A/K-12	O-:H37	R	18
P4 Δ*fim*	P4 Δ*fim*	A/K-12	O32:H37	S	This article
BEN2908	APEC	B2	O2:K1:H5	S	51
DM34	BEN2908 Δ*fim*	B2	O2:K1:H5	S	52
1303	MAEC	A/K-12	O5	S	53
K08	MAEC	A/R1	O48:H37	S	54
B117	ETEC	B1	O8:K85:K99	S mucoid	55, 56
A03	MAEC	B1/R4	O149	S	This article
BAS006	MAEC	A/R1	O15	S	This article
CEC5	MAEC	B1/R3	O54	S	This article
CEC11	MAEC	B1/R3	O139	S	This article
CEC21	MAEC	A/R1	O132	S	This article
DE6	MAEC	B1/R4	O1	S	This article

a MAEC, mastitis-associated E. coli; ETEC, enterotoxigenic E. coli; APEC, avian pathogenic E. coli.

### Assessment of deposition of antibodies onto bacteria by ELISA.

The binding of antibodies to OmpA or J5 bacteria to E. coli strains was assessed by ELISA as previously described (18). ELISA plates (96 flat-bottom wells; Nunc Immunosorp MaxiSorp) were coated with heat-killed (60°C for 30 min) bacteria. To improve the adherence of bacteria, the plates were coated with poly-l-lysine at 1 μg/ml for 2 h at ambient temperature. Bacterial suspensions (optical density at 620 nm [OD_620_] of 0.2 in PBS) were distributed (100 μl/well) and incubated overnight at 4°C. Finally, most of the PBS was removed and the plates were dried by incubation for 24 h at 37°C and stored at 4°C until use. The drying step proved to be essential for smooth strains to adhere to the wells, as smooth LPS (O-antigen) exerts a strong repulsion to polystyrene substrate (47). We checked by microscope examination that after rehydration and all the ELISA incubation and washing steps, a dense lawn of bacteria remained at the bottom of the wells. Nevertheless, some variation between strains occurred, with a trend for a denser bacterial lawn with rough strains.

The accessibility of OmpA to antibodies was assessed through the measurement of the binding of affinity-purified antibodies to recombinant E. coli OmpA by ELISA. The dilutions of antibodies were distributed in plates coated with the E. coli strain under test, in parallel with dilutions of PCCS, NBS, or sera of cows immunized with purified OmpA or E. coli J5 or P4 (18, 25).

### Adsorption of antisera with live bacteria.

Strains for adsorption were grown overnight in brain heart infusion broth (BHI) at 37°C. Overnight cultures were centrifuged 5 min at 2,500 × *g*, and bacteria were resuspended to an OD_600_ of 10 in Dulbecco’s PBS (DPBS) with Ca^2+^ (100 mg/liter CaCl_2_) and Mg^2+^ (100 mg/liter MgCl_2_-6H_2_O) supplemented with 0.1% bovine serum albumin (DPBSA+). In a 1.5-ml tube, 225 μl of the bacterial suspension was incubated with 25 μl of antisera to be depleted. Incubation was performed at 4°C with rolling agitation at 2 rpm for 1 h. The suspension was then centrifuged for 5 min at 2,500 × *g*, and the supernatant was collected and filtered on a 0.22-μm filter. The adsorbed antibody was then stored at –20°C.

### Assessment of the interaction of antibodies and complement with bacteria by flow cytometry.

We measured the binding of natural or vaccine-induced antibodies to live bacteria by using fluorophore-conjugated secondary antibodies and flow cytometry according to the principle described in reference 26. The bacteria from frozen stocks kept at –80°C were grown overnight in BHI. The bacteria (800 μl of culture) were centrifuged (4,000 × *g*, 20°C, 4 min), resuspended in DPBS with Ca^2+^ and Mg^2+^ supplemented with DPBSA+, centrifuged again, resuspended in 800 μl DPBSA+, and adjusted to about 5 × 10^7^ CFU/ml. Fifty microliters of this suspension was added to the antibody or serum dilution (500 μl), providing about 2.5 × 10^6^ bacteria per assay, and the mixtures were incubated for 45 min with occasional agitation in 1.5-ml snap-cap tubes (Eppendorf). The tubes then were centrifuged, the supernatant decanted, and 800 μl of DPBSA+ added, and the samples were centrifuged again and resuspended with 100 μl of secondary antibody, either anti-bovine IgG (H+L)-Alexa Fluor 647 at 1/200, anti-bovine IgM-fluorescein isothiocyanate (FITC), anti-bovine IgG2-FITC, or anti-bovine C3 MAb MD3 (Table 2). The tubes were incubated for 30 min on ice in the dark, centrifuged, and washed once, and the bacterial pellets were resuspended with 500 μl of DPBSA+. The tubes were kept on ice until analyzed by flow cytometry within 2 h after preparation. The acquisition of 20,000 events was carried out with a BD LSR Fortessa cytometer, and data were analyzed with Kaluza analysis software (Beckman Coulter). Gates were set up on the forward scatter (FSC)/side scatter (SCC) plot to remove debris and distinguish isolated from agglutinated bacteria.

**TABLE 2:** Primary and secondary antibodies used for flow cytometry analysis

Primary antibody	Secondary antibody (source)	Dilution
Bovine anti-OmpA	Goat anti-bovine IgG(H+L)-Alexa Fluor 647 (Jackson Immunoresearch)	1/200
Bovine serum	Sheep anti-bovine IgM-FITC (Bio-Rad Antibodies)	1/50
	Sheep anti-bovine IgG_2_-FITC (Bio-Rad Antibodies)	1/50
Mouse MAb to bovine C3 (clone MD3)	Rabbit F(ab′)2 a-mouse-IgG-RPE (Bio-Rad Antibodies)	1/100
Rabbit serum to type 1 fimbriae	Donkey anti-rabbit IgG-RPE (Jackson Immunoresearch)	1/200

### Assay of complement-dependent bactericidal activity.

Bacteria were grown in a blend (vol/vol) of DMEM/F-12–RPMI 1640 supplemented with 40 mM HEPES overnight, and a subculture in fresh medium was carried out for 3 h at 37°C. Bacteria were pelleted by centrifugation (1,500 × *g* for 5 min at 20°C) and resuspended in RPMI 1640 supplemented with 0.1% BSA and 20 mM HEPES (RPMI-AH). The assay was performed in sterile flat-bottomed 96-well microtiter plates by mixing 20 μl of bacteria at a concentration of 10^5^ CFU/ml with 180 μl of various concentrations of serum, either PCCS, PCCS plus antibodies, or NBS, and incubating for 3 h at 37°C. Survival was measured by plate count (spreading over a Trypticase soy agar [TSA] plate) after a series of 10-fold dilutions in DPBSA+. Mild sonication was applied to disperse the bacteria agglutinated by antibodies and conglutinin when NBS was used.

### Assay of phagocytic killing.

Bovine polymorphonuclear neutrophils (PMNs) were isolated from blood taken at the jugular vein in sterile evacuated tubes with EDTA. The tubes were centrifuged (1,000 × *g* for 10 min at 20°C), the plasma, the buffy coat, and the upper third of the red pellet were removed, and the red blood cells of the remaining pellet were lysed with lysing buffer (Sigma). PMNs were washed once with DPBS without Ca and Mg supplemented with RPMI-AH. The cells were adjusted to 2 × 10^6^/ml. Bacteria were grown overnight in BHI broth. They were washed once in DPBSA+ and resuspended in RPMI-AH at a concentration of 2 × 10^6^ CFU/ml. The phagocytic mixture comprised 250 μl of PMN suspension, 50 μl of bacterial suspension, and various amounts of antisera with or without PCCS as a source of complement. The mixture was adjusted to 0.5 ml with RPMI-AH in 1.5-ml Eppendorf tubes. The tubes were secured on a wheel at 37°C for 90 min and rotated (10 revolutions/min). At the end of the incubation, 50 μl of SDS (0.25% in DPBSA+) was added and the tubes were vortexed. After 1/10 dilution in DPBSA+, the CFU were enumerated by spreading 100 μl of the dilution onto TSA plates.

### Statistical analysis.

Statistical analyses were performed using GraphPad Prism 7.05 software, using tests for small size samples (see the figure legends). The statistical significance was considered a *P* value below 0.05.
