# Supplementary figures and images for: Shielding Effect of Escherichia coli O-Antigen Polysaccharide on J5-Induced Cross-Reactive Antibodies
Source: mSphere. 2021 Jan 27;6(1):e01227-20. doi: 10.1128/mSphere.01227-20 (PMC7885324; doi:10.1128/mSphere.01227-20)

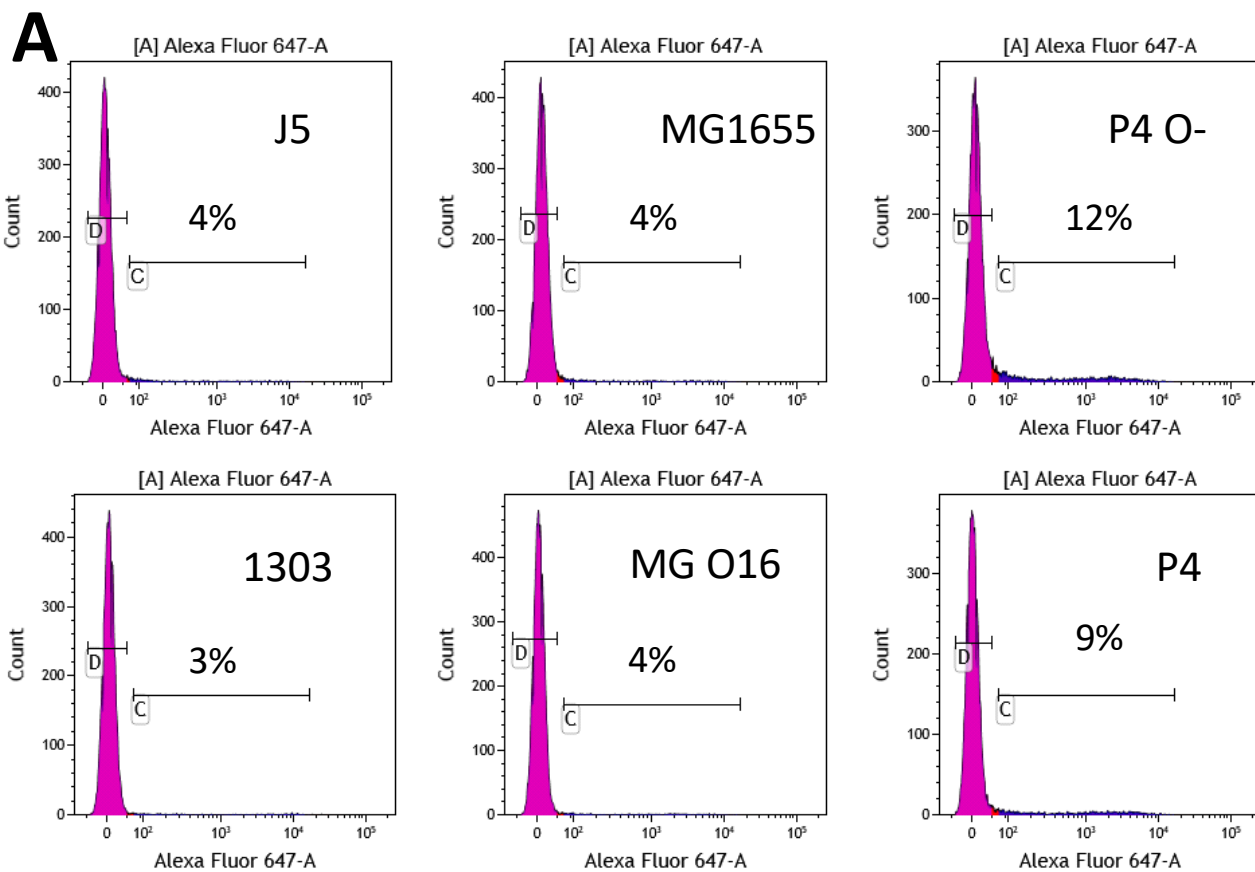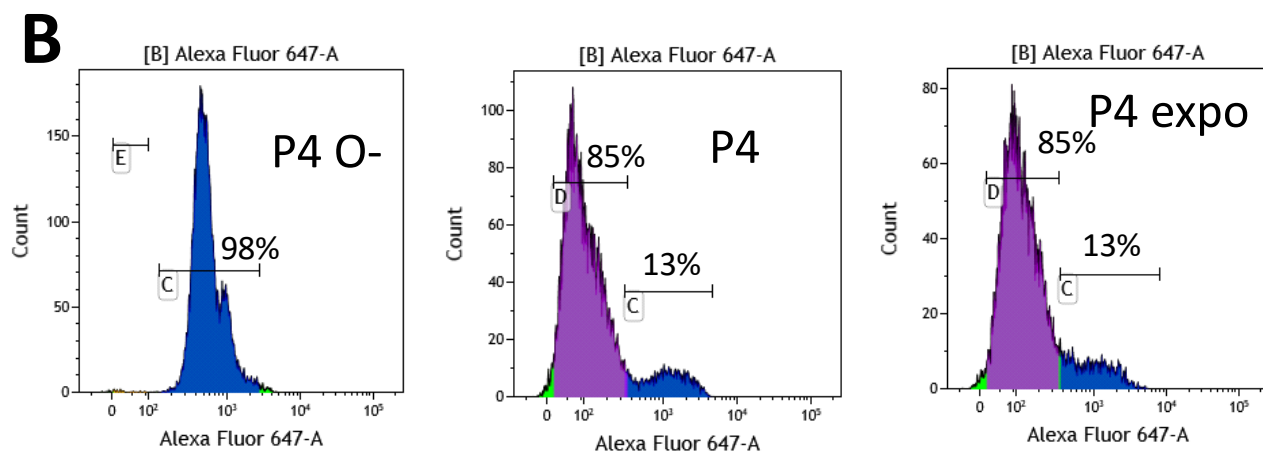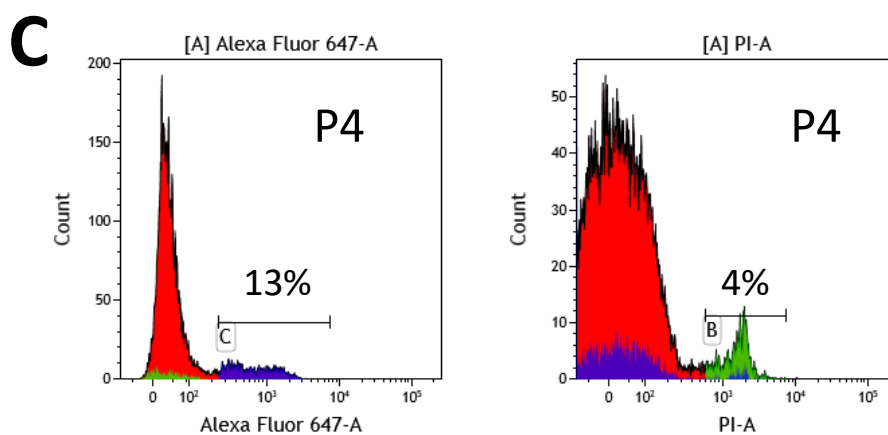

Supplement: FIG S1 [file mSphere.01227-20-sf001.pdf]

anti-OmpA 5 µg/mL

J5

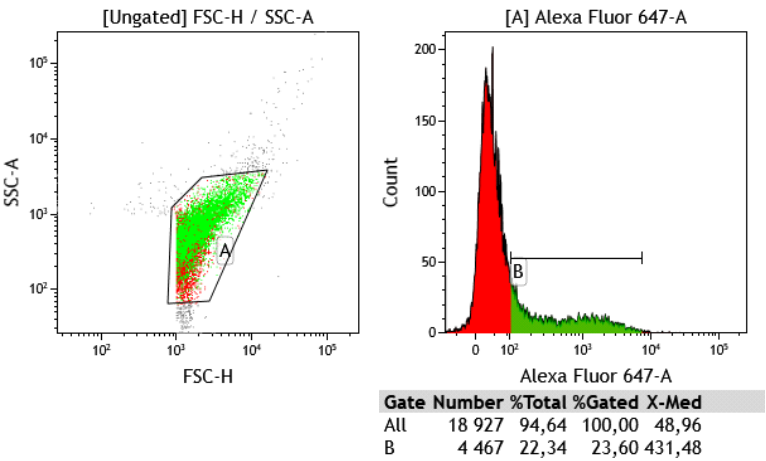

Supplement: FIG S2 [file mSphere.01227-20-sf002.pdf]

10% PCCS, 90 min

Anti- IgG & IgM

Anti-C3

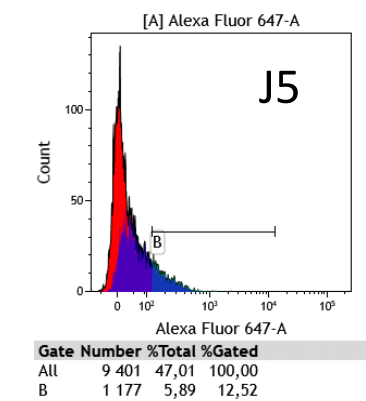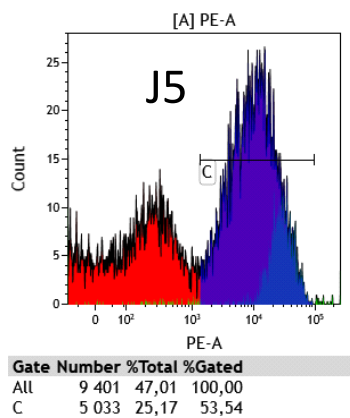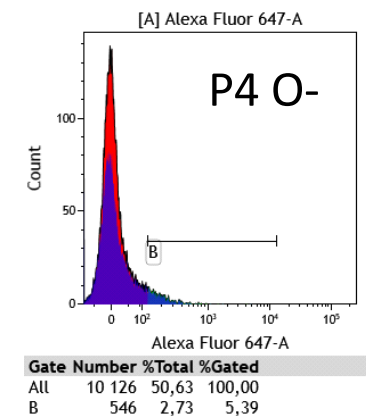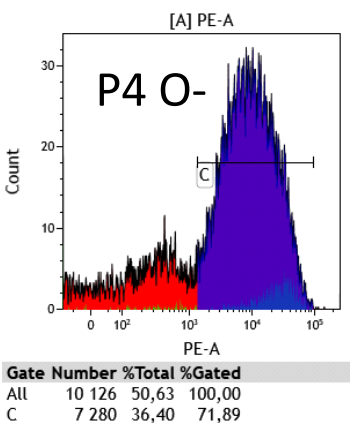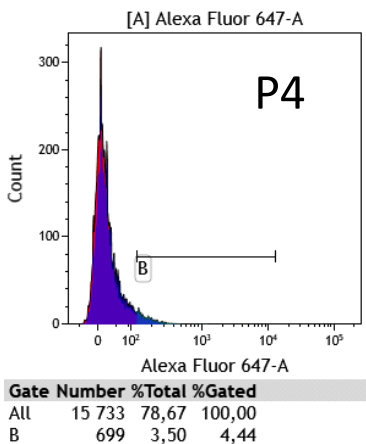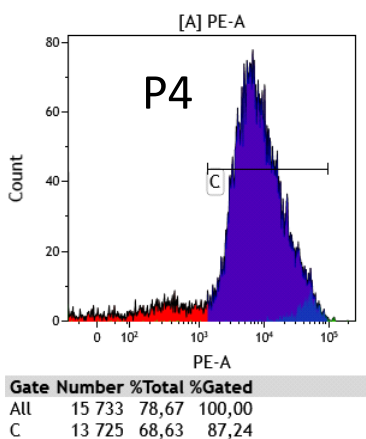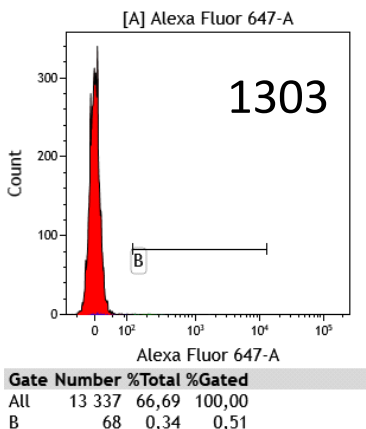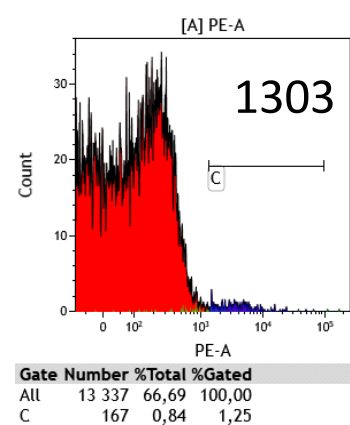

Supplement: FIG S4 [file mSphere.01227-20-sf004.pdf]
