# Supplementary material for: Shielding Effect of Escherichia coli O-Antigen Polysaccharide on J5-Induced Cross-Reactive Antibodies
Source: mSphere. 2021 Jan 27;6(1):e01227-20. doi: 10.1128/mSphere.01227-20 (PMC7885324; doi:10.1128/mSphere.01227-20)
Supplement: FIG S3 [file mSphere.01227-20-sf003.pdf]

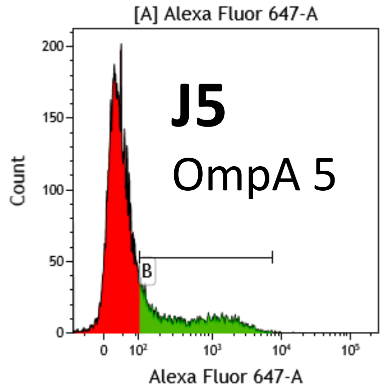

Gate	Number	%Total	%Gated	X-Med
All	18 674	93	100	48
B	4 282	21	23	406

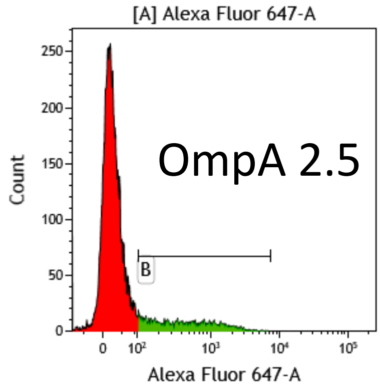

Gate	Number	%Total	%Gated	X-Med
All	18 784	94	100	27
B	2 742	14	15	437

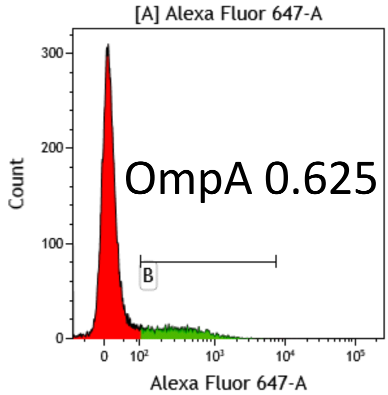

Gate	Number	%Total	%Gated	X-Med
All	18 763	94	100	16
B	2 584	13	14	332

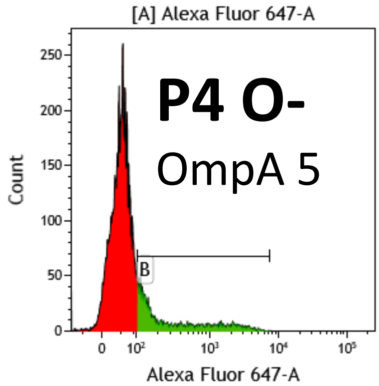

Gate	Number	%Total	%Gated	X-Med
All	18 599	93	100	60
B	3 710	19	20	229

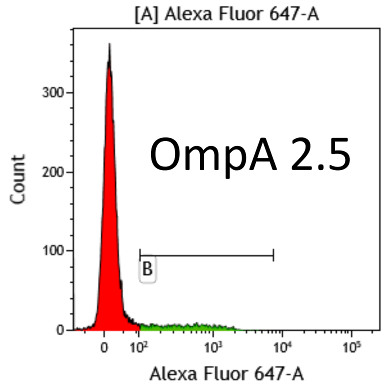

Gate	Number	%Total	%Gated	X-Med
All	18 709	94	100	17
B	1 856	9	10	443

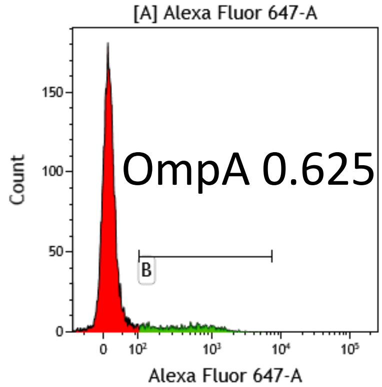

Gate	Number	%Total	%Gated	X-Med
All	9 170	92	100	17
B	975	10	11	458

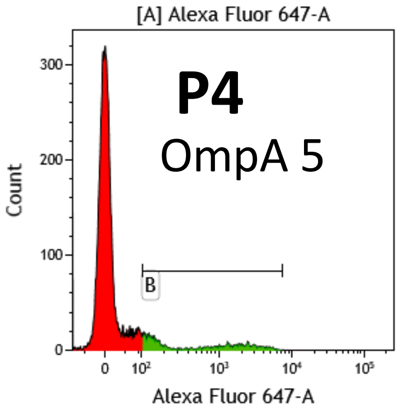

Gate	Number	%Total	%Gated	X-Med
All	17 380	87	100	4
B	2 271	11	13	585

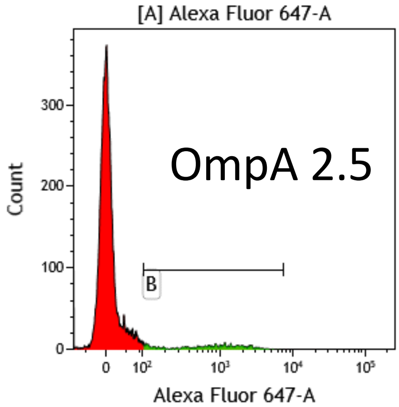

Gate	Number	%Total	%Gated	X-Med
All	17 796	89	100	4
B	1 364	7	8	836

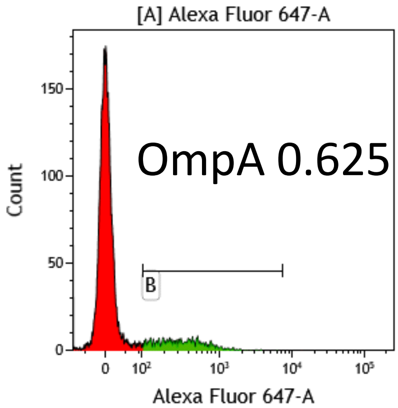

Gate	Number	%Total	%Gated	X-Med
All	8 804	88	100	4
B	1 200	12	14	327

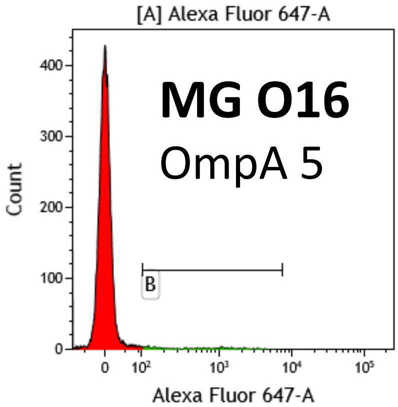

Gate	Number	%Total	%Gated	X-Med
All	18 645	93	100	1
B	874	4	5	548

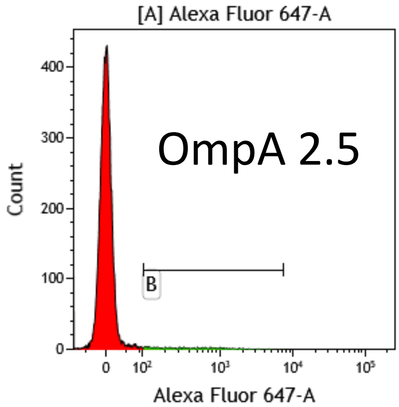

Gate	Number	%Total	%Gated	X-Med
All	18 888	94	100	1
B	843	4	4	439

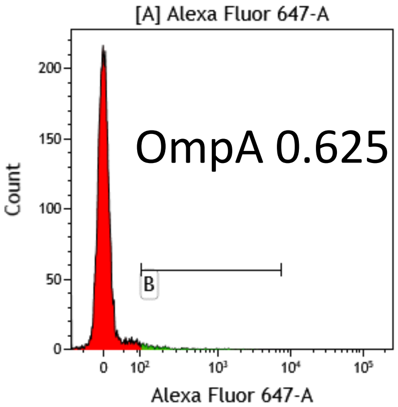

Gate	Number	%Total	%Gated	X-Med
All	9 508	95	100	1
B	313	3	3	202
