# Supplementary material for: Shielding Effect of Escherichia coli O-Antigen Polysaccharide on J5-Induced Cross-Reactive Antibodies
Source: mSphere. 2021 Jan 27;6(1):e01227-20. doi: 10.1128/mSphere.01227-20 (PMC7885324; doi:10.1128/mSphere.01227-20)
Supplement: TABLE S1 [file mSphere.01227-20-st001.docx]

Suppl. Table 1. Resistance of *E. coli* strains to the bactericidal activity of complement.

| **% PCCS** | **P4** | **P4 O-** | **B117** | **K08** | **MG1655** | **MG O16** |
| --- | --- | --- | --- | --- | --- | --- |
| **0** | 233 | 96 | 175 | 222 | 172 | 171 |
| **5** | >500 | 0 | >500 | >500 | 0 | >500 |
| **10** | >500 | 0 | >500 | >500 | 0 | >500 |
| **20** | >500 | 0 | >500 | >500 | 0 | >500 |
| **33** | >500 | 0 | 400 | >500 | ND | >500 |
| **50** | >500 | ND | 204 | >500 | ND | >500 |
| **80** | >500 | ND | 34 | >500 | ND | 400 |

Figures are numbers of cfu after 3h of incubation of bacteria at 37°C with the indicated final concentrations of serum. PCCS was used as a source of complement almost devoid of antibodies.

ND : not done.
