# Supplementary material for: Shielding Effect of Escherichia coli O-Antigen Polysaccharide on J5-Induced Cross-Reactive Antibodies
Source: mSphere. 2021 Jan 27;6(1):e01227-20. doi: 10.1128/mSphere.01227-20 (PMC7885324; doi:10.1128/mSphere.01227-20)
Supplement: TABLE S2 [file mSphere.01227-20-st002.docx]

Suppl Table 2. Resistance of smooth *E. coli* strains to the bactericidal activity of normal bovine serum.

| **%SBN 2009** | **P4** | **P4 sonic** | **K08** | **K08 sonic** | **MG O16** | **MG O16 sonic** |
| --- | --- | --- | --- | --- | --- | --- |
| **0** | 218 |  | 208 |  | 131 |  |
| **5** | 530 |  | 560 |  | 510 |  |
| **10** | 620 |  | 640 |  | 320 |  |
| **20** | 650 |  | 890 |  | 640 |  |
| **33** | 510 | 860 | 810 | 1050 | 530 | 580 |
| **50** | 430 | 850 | 900 | 900 | 330 | 560 |
| **80** | 202 | 570 | 900 | 900 | 88 | 305 |

Figures are numbers of cfu after 3h of incubation of bacteria at 37°C with the indicated final concentrations of serum. SBN was used as a source of complement and antibodies. Sonication was used to disperse the bacteria agglutined by the antibodies and conglutinin.
